# Multicenter prospective registration study of efficacy and safety of capsule endoscopy in Crohn’s disease in Japan (SPREAD-J study)

**DOI:** 10.1007/s00535-023-02017-3

**Published:** 2023-07-21

**Authors:** Toshiyuki Sakurai, Teppei Omori, Hiroki Tanaka, Takahiro Ito, Katsuyoshi Ando, Takeshi Yamamura, Sohachi Nanjjo, Satoshi Osawa, Teruyuki Takeda, Kenji Watanabe, Hiroto Hiraga, Shuji Yamamoto, Keiji Ozeki, Shinji Tanaka, Hisao Tajiri, Masayuki Saruta, the SPREAD-J group, Tsuyoshi Akutagawa, Nobuo Aoyama, Toshihiro Iguchi, Katsuya Endo, Motohiro Esaki, Haruhiko Ogata, Shiro Oka, Naoki Omiya, Takeshi Kagaya, Kazuki Kakimoto, Taku Kobayashi, Shintaro Sagami, Hirotake Sakuraba, Shinichiro Shinzaki, Ken Sugimoto, Masaki Takao, Takehiro Torisu, Atsushi Nakajima, Konosuke Nakamichi, Masanao Nakamura, Hiroshi Nakase, Ryuhei Nishiyama, Yukie Hayashi, Mari Hayashida, Sakiko Hiraoka, Mikihiro Fuijya, Norimasa Fukada, Masayuki Fukuda, Hiroshi Mihara, Kaoru Yokoyama, Yuicihiro Yoshida, Kohei Wagatsuma

**Affiliations:** 1 Division of Gastroenterology and Hepatology, Department of Internal Medicine, The Jikei University School of Medicine, 3-25-8, Nishi-shimbashi, Minato-ku, Tokyo, 105-8461 Japan; 2 Institute of Gastroenterology, Tokyo Women’s Medical University, 8-1 Kawada-cho, Shinjuku-ku, Tokyo, 162-8666 Japan; 3 Sapporo IBD Clinic, Yamahana Doctor Town F2, 1-18, Minami-19, Nishi-8, Chuo-ku, Sapporo, Hokkaido 064-0919 Japan; 4 Inflammatory Bowel Disease Center, Sapporo Higashi Tokushukai Hospital, N33-E14, Higashi-ku, Sapporo, Hokkaido 065-0033 Japan; 5 Gastroenterology and Endoscopy, Division of Metabolism and Biosystemic Science, Gastroenterology and Hematology/Oncology, Department of Medicine, Asahikawa Medical University, 1-1-1, Higashi-nizyo, Midorigaoka, Asahikawa, Hokkaido 078-8510 Japan; 6 Department of Gastroenterology and Hepatology, Nagoya University Graduate School of Medicine, 65 Tsurumai-cho, Showa-ku, Nagoya, 466-8550 Japan; 7 Third Department of Internal Medicine, University of Toyama, 2630 Sugitani, Toyama, Toyama 930-0194 Japan; 8 Department of Endoscopic and Photodynamic Medicine, Hamamatsu University School of Medicine, 1-20-1, Handayama, Higashi-ku, Hamamatsu, 431-3192 Japan; 9 Inflammatory Bowel Disease Center, Fukuoka University Chikushi Hospital, 1-1-1, Zokumyoin, Chikushino, Fukuoka 818-8502 Japan; 10 Division of Gastroenterology and Hepatology, Department of Internal Medicine, Hyogo Medical University, 1-1, Mukogawa-cho, Nishinomiya, Hyogo 663-8501 Japan; 11 Department of Gastroenterology and Hematology, Hirosaki University Graduate School of Medicine, 53-Hon-cho, Hirosaki, Aomori 036-8563 Japan; 12 Department of Gastroenterology and Hepatology, Graduate School of Medicine, Kyoto University, 54 Shogoin-Kawahara-cho, Sakyo-ku, Kyoto, 606-8507 Japan; 13 Department of Gastroenterology and Metabolism, Nagoya City University Graduate School of Medical Sciences, 1, Kawasumi, Mizuho-machi, Mizuho-ku, Nagoya, 467-0001 Japan; 14 Department of Endoscopy, Hiroshima University Hospital, Hiroshima, 1-2-3, Kasumi, Minami-ku, Hiroshima, 734-8551 Japan; 15 Department of Innovative Interventional Endoscopy Research, The Jikei University School of Medicine, 3-25-8, Nishi-shimbashi, Minato-ku, Tokyo, 105-8461 Japan

**Keywords:** Small-bowel capsule endoscopy, Crohn’s disease, Efficacy, Safety, Prospective study

## Abstract

**Background:**

Evidence of small-bowel capsule endoscopy (SBCE) for evaluating lesions in Crohn’s disease (CD) is lacking. We aimed to clarify the effectiveness and safety of SBCE in a large sample of patients with CD.

**Methods:**

This multicenter prospective registration study recorded the clinical information and SBCE results of patients with definitive CD (d-CD) or suspected CD (s-CD). The primary outcomes were the rates of successful assessment of disease activity using SBCE, definitive diagnosis of CD, and adverse events. Secondary outcomes were the assessment of SBCE findings in patients with d-CD and s-CD and factors affecting SBCE incompletion and retention; and tertiary outcomes included the association between clinical disease activity or blood examination, endoscopic disease activity, ileal CD, and the questionnaire assessment of patient acceptance of SBCE.

**Results:**

Of 544 patients analyzed, 541 underwent SBCE with 7 (1.3%) retention cases. Of 468 patients with d-CD, 97.6% could be evaluated for endoscopic activity. Of 76 patients with s-CD, 15.8% were diagnosed with ‘confirmed CD’. CD lesions were more frequently observed in the ileum and were only seen in the jejunum in 3.4% of the patients. Male sex and stenosis were risk factors for incomplete SBCE, and high C-reactive protein levels and stenosis were risk factors for capsule retention. In L1 (Montreal classification) patients, clinical remission was associated with endoscopic remission but showed low specificity and accuracy. The answers to the acceptability questionnaire showed the minimal invasiveness and tolerability of SBCE.

**Conclusion:**

SBCE is practical and safe in patients with CD.

**Supplementary Information:**

The online version contains supplementary material available at 10.1007/s00535-023-02017-3.

## Introduction

Crohn’s disease (CD), is an inflammatory bowel disease with unknown pathogenesis that causes irreversible intestinal damage, such as stenosis, perforation, and fistula. Repeated irreversible damage necessitates intestinal resection, and 80% of patients with CD require surgical treatment throughout the course of the disease [1, 2]. Approximately 80% of patients with CD have lesions in the small intestine, and 30% of patients with CD have lesions only in the small intestine [1, 2]. Endoscopic healing (EH) is described as the ideal goal for patients with inflammatory bowel disease (IBD) in the Selecting Therapeutic Targets in Inflammatory Bowel Disease (STRIDE)-II [3] initiative, which states that symptomatic response is the first target; symptomatic remission and normalization of C-reactive protein (CRP) are the second targets; decrease in calprotectin to an acceptable range is the third target; and EH is the final target. EH is the most favorable condition in patients with CD to predict a surgery-free prognosis [4].

Therefore, patients with CD need an evaluation of the entire small intestine, not only to diagnose or assess disease activity before and after treatment but also to confirm the maintenance of EH. The most reliable way to assess the condition of the small intestinal mucosa is to observe it directly using endoscopy; however, balloon-assisted endoscopy (BAE) is invasive. Thus, less invasive examination tools, such as magnetic resonance enterography (MRE), computed tomography enterography (CTE), abdominal ultrasound, and small-bowel capsule endoscopy (SBCE), have been performed.

SBCE is noninvasive, can facilitate examination of the entire small intestine in a single inspection, and can detect tiny lesions, such as aphthae or denudation, which are difficult to detect using BAE or cross-sectional imaging [5, 6]. The major disadvantage is the incompletion or retention of SBCE in patients with severe stenosis of the small intestine [7, 8]. To prevent such adverse events, a pre-confirmatory patency capsule (PC) test is usually performed before SBCE.

Although many positive reports have been published on the usefulness and safety of SBCE in patients with CD [5, 9–12], the level of evidence was not high in those reports because of the small sample sizes, except for a few meta-analyses [5]. The lack of a gold standard for the definitive diagnosis of CD makes it challenging to evaluate the effectiveness of SBCE. Similarly, there are few large-sample reports on the safety of SBCE, including the prevalence of retention [7, 8, 13]. Thus, IBD guidelines describe SBCE as a monitoring tool with weak recommendations and insufficient evidence [14–16]. The Japanese IBD guidelines also state that “capsule endoscopy may be useful” in the medical treatment of CD [17]. In addition, there are a few reports on the efficacy of SBCE for monitoring endoscopic disease activity.

Although the lack of a gold standard makes it difficult to establish evidence for the assessment of small-intestinal findings of CD, a study with a large sample size is required. Therefore, this study aimed to evaluate the efficacy and safety of SBCE for the medical treatment of CD using a large sample.

## Methods

We conducted a multicenter prospective registration study involving 45 institutes in Japan. Regardless of sex, patients aged 16–80 years who were scheduled to undergo SBCE to evaluate definitive CD (d-CD) or obtain a definitive diagnosis in patients with suspected CD (s-CD) from October 2018 to April 2021 were considered for enrollment non-consecutively in this study. Patients were excluded if they had a gastrointestinal obstruction or fistula, no gastrointestinal patency, dysphagia, a cardiac pacemaker, any electronic medical device, or were pregnant.

After obtaining written informed consent and performing SBCE, the attending physician in each institute registered the pertinent information on the registration site in the Center for Clinical Research of Hamamatsu University School of Medicine. The information included the following: patient’s clinical information (sex, age, disease phenotype, disease period, the purpose of examination, concomitant drug use, blood sampling data, and CD activity index [CDAI] [18]), information related to the PC test (whether to implement the PC test, the ratio of confirmed patency, and adverse events), SBCE results (findings of SBCE, capsule endoscopy Crohnʼs disease activity index [CECDAI] [19], Lewis score [LS] [20], and adverse events), and answers to the questionnaire on patient acceptance.

The primary outcomes were the rates of successful assessment of disease activity by SBCE, definitive diagnosis of CD, and adverse events. We compared the findings of SBCE with those of other modalities such as balloon-assisted endoscopy (BAE), computed tomography enterography (CTE), and magnetic resonate imaging enterography (MRE) if those modalities were used at the same time. The secondary outcomes were the assessment of SBCE findings in patients with d-CD and s-CD and factors affecting the incompletion and retention of SBCE. The tertiary outcomes were the correlation and association between clinical disease activity or blood examination and endoscopic disease activity in patients with the ileal type (L1 of Montreal classification [21]) of d-CD and the assessment questionnaire on patient acceptance of SBCE.

### Analysis of primary outcomes

We asked the attending physician to register whether it was possible to evaluate the disease activity of CD from SBCE findings in patients with d-CD. Similarly, we asked the attending physician to register if it was possible to obtain a definitive diagnosis from SBCE findings in patients with s-CD. We calculated the success ratio for assessing the endoscopic activity of d-CD and obtaining a definitive diagnosis in patients with s-CD. Additionally, we calculated the frequency and details of the adverse events associated with SBCE. Further, we compared SBCE findings with BAE, CTE, and MRE if performed within 1 month before or after the day of performing SBCE.

### Analysis of secondary outcomes

The ratios of findings such as edema, aphtha, erosion, ulcers, longitudinal ulcers, cobblestone appearance, and stenosis observed in the jejunum and ileum were calculated in patients with d-CD (all phenotypes and L1 of Montreal classification only) and s-CD. In addition, we investigated the findings that contributed to the diagnosis of ‘confirmed CD’ and ‘suspicious CD’ in Japanese diagnostic criteria [17, 22, 23] in patients with s-CD. Furthermore, we analyzed the factors associated with the incompletion and retention of SBCE.

### Analysis of tertiary outcomes

The relationship between clinical remission, defined as CDAI < 150 or CRP < 0.15 mg/dL, and endoscopic remission, defined as CECDAI < 3.5 [24] or LS < 135 [20], was analyzed in L1 patients with a Montreal classification of d-CD. Sensitivity, specificity, positive predictive value (PPV), negative predictive value (NPV), and accuracy were calculated. Finally, we determined the receiver operating characteristics (ROC) curve of CDAI and CRP for the determination of CECDAI < 3.5 or LS < 135 only in L1 patients according to the Montreal classification. We further evaluated the ratio of successful evaluation among three grades of CD activity based on CDAI as remission (< 150), mild (≥ 150, < 220), and moderate (≥ 220, < 450). Finally, we analyzed the proportion of acceptance of SBCE in the questionnaire for all participants.

### CD diagnostic criteria

The definitive diagnosis of CD was based on the diagnostic criteria used in Japan [17, 22, 23], as shown in Supplementary Table 1. If the findings do not fulfill ‘confirmed CD,’ the diagnosis is ‘suspicious CD’ in Japan.

### Small bowel capsule endoscopy

The method of performing SBCE was the same for all the facilities. In a state of fasting for 12 h or more, the capsule endoscope was orally ingested, water intake was started 2 h later, and a meal was eaten 4 h later. All institutes used PillCam (Medtronic, Dublin, Ireland), and the SBCE results were interpreted using the PillCam RAPID Reader (Medtronic) by an interpretation doctor at each facility.

### Definition of capsule endoscopic findings

We defined aphthae as a ‘diminutive loss of epithelial layering with a whitish center and a red halo’; erosion, ‘mucosal break < 5 mm’; and ulceration, ‘mucosal break > 5 mm.’ We also defined longitudinal ulcers as ‘linear ulcers that run longitudinally along with the gastrointestinal tract’ and stenosis as ‘narrowing intestinal lumens withholding the passage of the capsule endoscope.’

### Patency capsule test

The same PC test method was used for each facility. The PillCam PC (Medtronic, Dublin, Ireland) was also orally ingested in a fasted state of at least 12 h or more. From 20 to 30 h later, whether the PC was excreted from the anus intact, or whether the PC reached the large intestine was confirmed by radiography, abdominal computed tomography, abdominal ultrasound, or tomosynthesis.

### The questionnaire on patient acceptance of SBCE

The questionnaire on patient acceptance of SBCE consisted of six questions that asked (1) whether they felt embarrassment, fear, pain, and difficulties in swallowing the capsule during SBCE; (2) which did they think was more accessible than SBCE; and (3) were they willing to proceed with SBCE next time.

### Statistical analysis

Factors associated with the successful evaluation of endoscopic disease activity and completion of SBCE were analyzed using the Chi-square test or multiple regression analysis, and multivariate analysis was performed using logistic regression analysis. The comparison of a successful evaluation of endoscopic disease activity with other modalities was analyzed using McNemar’s test. The relationship between CDAI < 150 and CRP < 0.15 mg/dL with CECDAI < 3.5 or LS < 135 was analyzed using McNemar’s test. The relationship between CRP levels and other factors was analyzed using a *t* test or Wilcoxon rank-sum test. The comparison of a successful evaluation among three grades of CD activity was analyzed using a Chi-square test. Statistical significance was set at *P* < 0.05. Data are presented as mean ± standard deviation or median with interquartile range. All statistical analyses were performed using STATA ver. 15.0 (Stata Corp, College Station, TX, USA).

### Ethical approval

This study was approved by the Jikei Hospital Ethics Committee (No. 8451) and conducted in accordance with the provisions of the Declaration of Helsinki. All participants provided written informed consent prior to participating in the study. This study was registered with the UMIN Clinical Trials Registry (No. 000037143).

## Results

In total, 558 inspections were enrolled and registered from 34 institutes in Japan. No inspections were registered from 11 institutes. Of these, 14 misregistered cases were excluded; thus, 544 inspections were eligible for the analysis. Approximately 70% of the patients were male, and the mean age was 34.8 ± 13.5 years. Eighty-six percent of eligible patients had d-CD. The mean CDAI was 89.2 ± 72.7, and the median CRP was 0.1 mg/dL (IQR 0.03–0.32). The patient characteristics are shown in Supplementary Table 2.

The PC test was performed in 90.8% of the patients, and only three were without patency. No adverse events were observed during the PC test. SBCE was performed in 541 patients: 491 patients with confirmed patency and 50 patients who did not undergo a PC test (Supplementary Fig. 1). Incompletion of SBCE was observed in 35 cases (6.5%), with only seven retention cases (1.3%). The median LS and CECDAI were 135 (IQR 0–337) and 4 (IQR 0–9), respectively.

### Analysis of primary outcomes

Endoscopic disease activity was evaluated in 97.6% of patients with d-CD. Of 76 patients with s-CD, 44 (57.9%) showed findings related to CD (Table 1). There were only seven adverse events (1.3%), all of which were the retention of the capsule endoscope with no need for surgical treatment.

**Table 1:** Results of SBCE and PC test

	Total	d-CD	s-CD	*P* value
Number of subjects, *N*	544	468	76	
Implemented PC test, *n* (%)	494 (90.8)	429 (91.7)	65 (85.5)	0.086
Patency confirmed, *n* (%)	491 (99.4)	426 (99.3)	65 (100.0)	0.499
Adverse event of SBCE, *n* (%)	7 (1.3)	6 (1.3)	1 (1.3)	0.985
Retention, *n* (%)	7 (1.3)	6 (1.3)	1 (1.3)	0.985
Completion of SBCE, *n* (%)	506 (93.5)	432 (92.3)	74 (97.4)	0.142
Success evaluation of disease activity, *n* (%)		457 (97.6)		N.A.
Obtaining confirmed diagnosis, *n* (%)			44 (57.9)	N.A.
Confirmed CD			12 (15.8)	N.A.
Suspected CD			32 (42.1)	N.A.
Exclusion of CD			32 (42.1)	N.A.
CECDAI, median (IQR)		4 (0–9)		N.A.
LS, median (IQR)		135 (0–337)		N.A.

*d-CD* definitive Crohn’s disease, *s-CD* suspected Crohn’s disease, *PC* patency capsule, *SBCE* small-bowel capsule endoscopy, *CECDAI* capsule endoscopy Crohn’s disease activity index, *LS* Lewis score, *IQR* interquartile range, *N.A.* not applicable

Of 541 patients who underwent SBCE, BAE was performed in 15 patients and MRE was performed in 8 patients. None of the cases performed CTE. The rate to detect findings of lesions of CD and successful evaluation of mucosal lesions of SBCE and BAE were equivalent, except for a trend of discrepancy in the detection of longitudinal ulcers. In contrast, those of SBCE and MRE were different. In particular, SBCE was superior to MRE for the detection of aphtha or erosion (Table 2).

**Table 2:** A comparison of diagnostic yield of (a) SBCE with BAE, and (b) SBCE with MRE

(a) *N* = 15	SBCE													
	Aphtha/erosion		Ulcer		Longitudinal ulcer		Cobblestone appearance		Stenosis		Successful evaluation		Diagnosed with d-CD	
	+	−	+	−	+	−	+	−	+	−	+	−	+	−
BAE														
+	7	3	7	3	4	3	0	3	4	0	10	0	3	0
−	3	2	3	2	0	8	0	12	2	9	1	0	0	1
*P* value	0.655		0.655		0.083		0.083		0.157		0.317		N.A	

(b) *N* = 8	SBCE													
	Aphtha/erosion		Ulcer		Longitudinal ulcer		Cobblestone appearance		Stenosis		Successful evaluation		Diagnosed with d-CD	
	+	−	+	−	+	−	+	−	+	−	+	−	+	−
MRE														
+	0	0	2	1	0	1	0	0	0	0	3	0	2	0
−	7	1	3	2	5	2	0	8	1	7	3	0	0	0
*P* value	0.008		0.317		0.103		N.A.		0.317		0.083		N.A.	

*BAE* balloon-assisted endoscopy, *d-CD* diagnosed Crohn’s disease, *N.A.* not applicable, *SBCE* small intestinal capsule endoscopy

### Analysis of secondary outcomes

The details of the SBCE findings are presented in Table 3. CD lesions were more frequently detected in the ileum than in the jejunum (*P* < 0.001), including all types of inflammatory findings. CD lesions were seen in the ileum in approximately 70% and 65% of the d-CD and L1 patients, respectively. CD findings were seen only in the ileum and jejunum in 31.2% and 3.4% of patients with d-CD, respectively.

**Table 3:** Findings of SBCE in patients with A) d-CD, B) L1 patients with d-CD, and C) s-CD

	d-CD			L1 with d-CD			s-CD	
	Jejunum	Ileum	*P* value	Jejunum	Ileum	*P* value	Jejunum	Ileum
Number of subjects, *N*	443	439		102	102		76	74
Edema, *n* (%)	50 (11.3)	82 (18.7)	0.003	19 (18.6)	23 (22.5)	0.403	13 (17.1)	20 (27.0)
Aphtha, *n* (%)	71 (16.0)	117 (26.7)	< 0.001	18 (17.6)	24 (23.5)	0.303	11 (14.5)	17 (23.0)
Erosion, *n* (%)	106 (23.9)	171 (39.0)	< 0.001	16 (15.7)	34 (33.3)	0.002	22 (28.9)	27 (36.5)
Ulcer, *n* (%)	48 (10.8)	126 (28.7)	< 0.001	12 (11.8)	35 (34.3)	< 0.001	10 (13.2)	18 (24.3)
Longitudinal ulcer, *n* (%)	10 (2.3)	31 (7.1)	< 0.001	2 (2.0)	7 (6.9)	0.122	4 (5.3)	8 (10.8)
Cobblestone appearance, *n* (%)	2 (0.5)	8 (1.8)	0.056	0 (0.0)	2 (2.0)	0.155	0 (0.0)	1 (1.4)
Stenosis, *n* (%)	13 (2.9)	71 (16.1)	< 0.001	5 (4.9)	20 (19.6)	0.001	2 (2.6)	5 (6.8)
Disease location in small intestine, *n* (%)								
Jejunum alone, *n* (%)	15 (3.4)		< 0.001	6 (5.9)		0.002	7 (9.2)	
Ileum alone, *n* (%)		137 (31.2)			34 (33.3)			18 (24.3)
No findings of CD, *n* (%)	138 (29.5)			28 (27.5)			26 (34.2)	

*d-CD* definitive Crohn’s disease, *s-CD* suspected Crohn’s disease

**Fig. 1:**
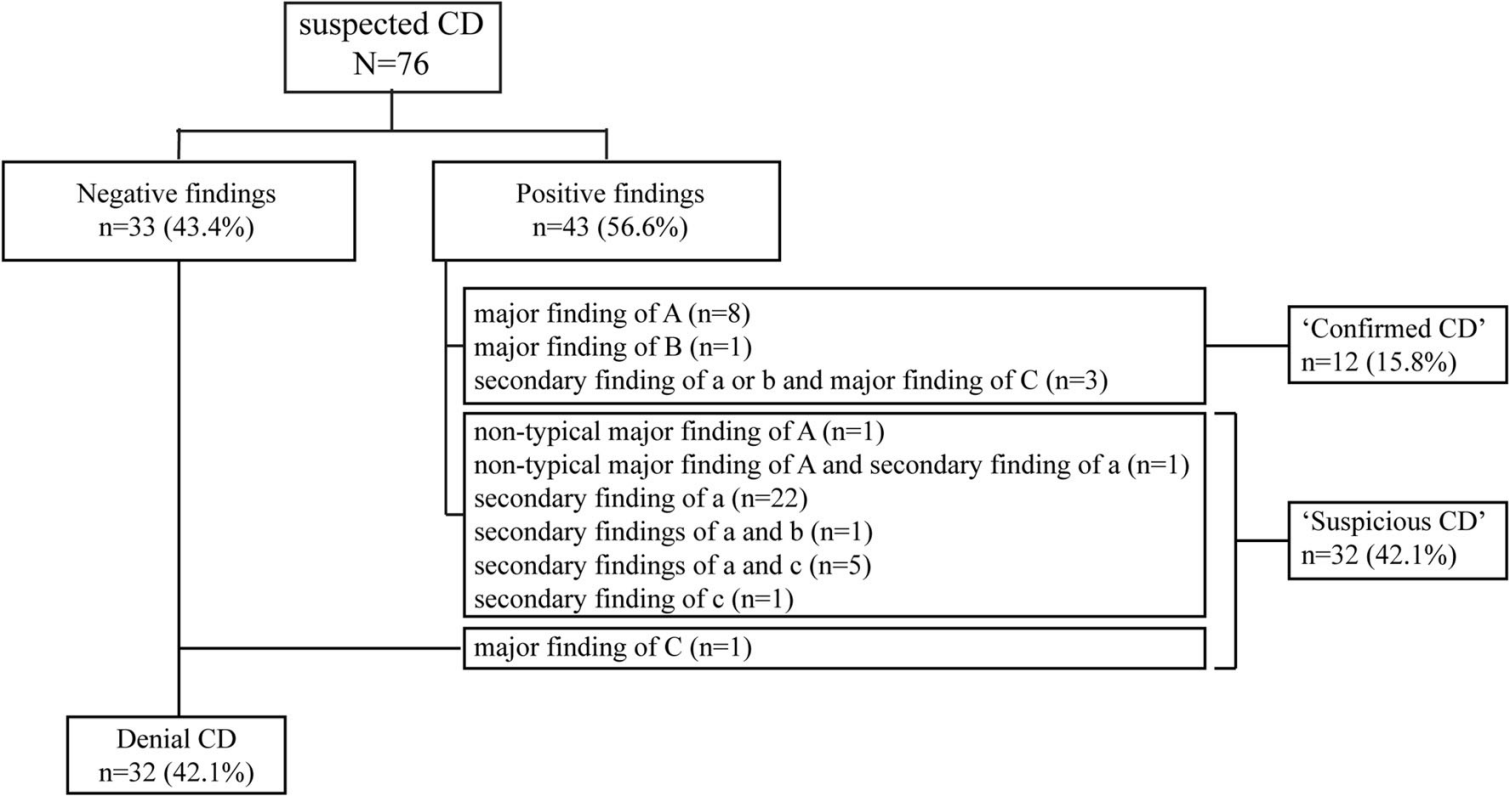
Diagnostic flow diagram of patients with suspected CD. Of the 76 patients with s-CD, 12 and 32 were diagnosed with d-CD and s-CD, respectively, and CD was excluded in 32 patients. *CD* Crohn’s disease, *s-CD* suspected Crohn’s disease, *d-CD* definitive Crohn’s disease

Of 76 patients with s-CD, 12 (15.8%) were diagnosed with d-CD based on the findings of longitudinal ulcers (*n* = 8), cobblestone appearance (*n* = 1), and secondary findings and granulomas (*n* = 3). Thirty-two (42.1%) patients were diagnosed with s-CD using the Japanese diagnostic criteria, and CD was excluded in 32 (42.1%) patients (Fig. 1). Three of the 32 patients with ‘suspicious CD’ were later diagnosed with ‘confirmed CD’ in combination with the results of other modalities. A further 6 patients were subsequently diagnosed with other diseases, and the remaining 23 cases remained ‘suspicious CD.’ Factors affecting incompletion of SBCE were male sex (OR 6.6, 95% CI 1.5–29.1) and the presence of stenosis (OR 4.7, 95% CI 2.1–10.5). Factors affecting SBCE retention were CRP elevation (OR 3.9, 95% CI 1.5–10.4) and the presence of stenosis (OR 521.4, 95% CI 4.5–60,869.2) (Table 4).

**Table 4:** An analysis of factors associated with (A) incompletion of SBCE and (B) retention of SBCE

(A) Incompletion of SBCE				
Factors	Completion (*N* = 506)	Incompletion (*N* = 35)	*P* value	Multivariate analysis
Sex, male, *n* (%)	345 (68.2)	33 (94.3)	0.001	6.6 (95% CI 1.5–29.1)
Age, mean ± SD	34.4 ± 13.4	40.4 ± 14.2	0.006	1.0 (95% CI 1.0–1.1)
History of intestinal resection, *n* (%)	140 (27.7)	12 (34.3)	0.412	
Disease period, months, median (IQR)	73 (27–147)	133 (48–215)	0.011	1.0 (95% CI 1.0–1.0)
Proceeding PC test, *n* (%)	458 (90.5)	33 (94.3)	0.456	
WBC, /μL, mean ± SD	6217 ± 1981	5597 ± 2229	0.038	1.0 (95% CI 1.0–1.1)
Hb, g/dL, mean ± SD	13.7 ± 1.9	13.8 ± 1.9	0.567	
PLT, 10^4^/μL, mean ± SD	27.6 ± 7.9	25.3 ± 8.2	0.048	1.0 (95% CI 0.9–1.1)
ESR, mm, median (IQR)	11 (5–24)	11 (4.5–17)	0.546	
Albumin, g/dL, mean ± SD	4.2 ± 0.5	4.3 ± 0.5	0.433	
CRP, mg/dL, median (IQR)	0.1 (0.03–0.31)	0.13 (0.03–0.37)	0.541	
TC, mg/dL, mean ± SD	164 ± 37	167 ± 28	0.422	
CDAI, mean ± SD	89.1 ± 72.7	93.0 ± 76.1	0.777	
Ulcer, *n* (%)	147 (29.1)	12 (41.4)	0.158	
Longitudinal ulcer, *n* (%)	41 (8.1)	1 (3.4)	0.365	
Stenosis, *n* (%)	66 (13.0)	13 (37.1)	< 0.001	4.7 (95% CI 2.1–10.5)

(B) Retention of SBCE				
Factors	Retention (*N* = 7)	No-retention (*N* = 534)	*P* value	Multivariate analysis
Sex, male, *n* (%)	7 (100.0)	371 (69.5)	0.001	1
Age, mean ± SD	30.7 ± 10.0	34.9 ± 13.6	0.423	
History of intestinal resection, *n* (%)	2 (28.6)	150 (28.1)	0.985	
Disease period, months, median (IQR)	105.1 ± 88.9	109.6 ± 107.8	0.913	
Proceeding PC test, *n* (%)	6 (85.7)	485 (90.8)	0.643	
WBC, /μL, mean ± SD	5718 ± 1700	6182 ± 2006	0.543	
Hb, g/dL, mean ± SD	12.3 ± 3.1	13.7 ± 1.8	0.051	
PLT, 10^4^/μL, mean ± SD	33.7 ± 14.8	27.4 ± 7.8	0.036	0.9 (95% CI 0.8–1.1)
ESR, mm, median (IQR)	55 (7–56)	11 (5–22)	0.181	
Albumin, g/dL, mean ± SD	3.9 ± 0.9	4.3 ± 0.5	0.048	2.2 (95% CI 0.2–31.1)
CRP, mg/dL, median (IQR)	0.47 (0.19–6.63)	0.1 (0.03–0.31)	< 0.01	3.9 (95% CI 1.5–10.4)
TC, mg/dL, mean ± SD	131.7 ± 23.4	164.9 ± 36.6	0.117	
CDAI, mean ± SD	125.8 ± 114.4	88.8 ± 72.1	0.183	
Ulcer, *n* (%)	4 (57.1)	155 (29.0)	0.046	0.4 (95% CI 0.0–4.8)
Longitudinal ulcer, *n* (%)	1 (14.3)	41 (7.7)	0.419	
Stenosis, n (%)	5 (71.4)	74 (13.9)	< 0.001	521.4 (95% CI 4.5.–60,869.2)

*CI* confidence interval, *SD* standard deviation, *IQR* interquartile range, *WBC* white blood cell, *Hb* hemoglobin, *PLT* platelet, *ESR* erythrocyte sedimentation rate, *CRP* C-reactive protein, *TC* total cholesterol, *CDAI* Crohn’s disease activity index

### Analysis of tertiary outcomes

**Fig. 2:**
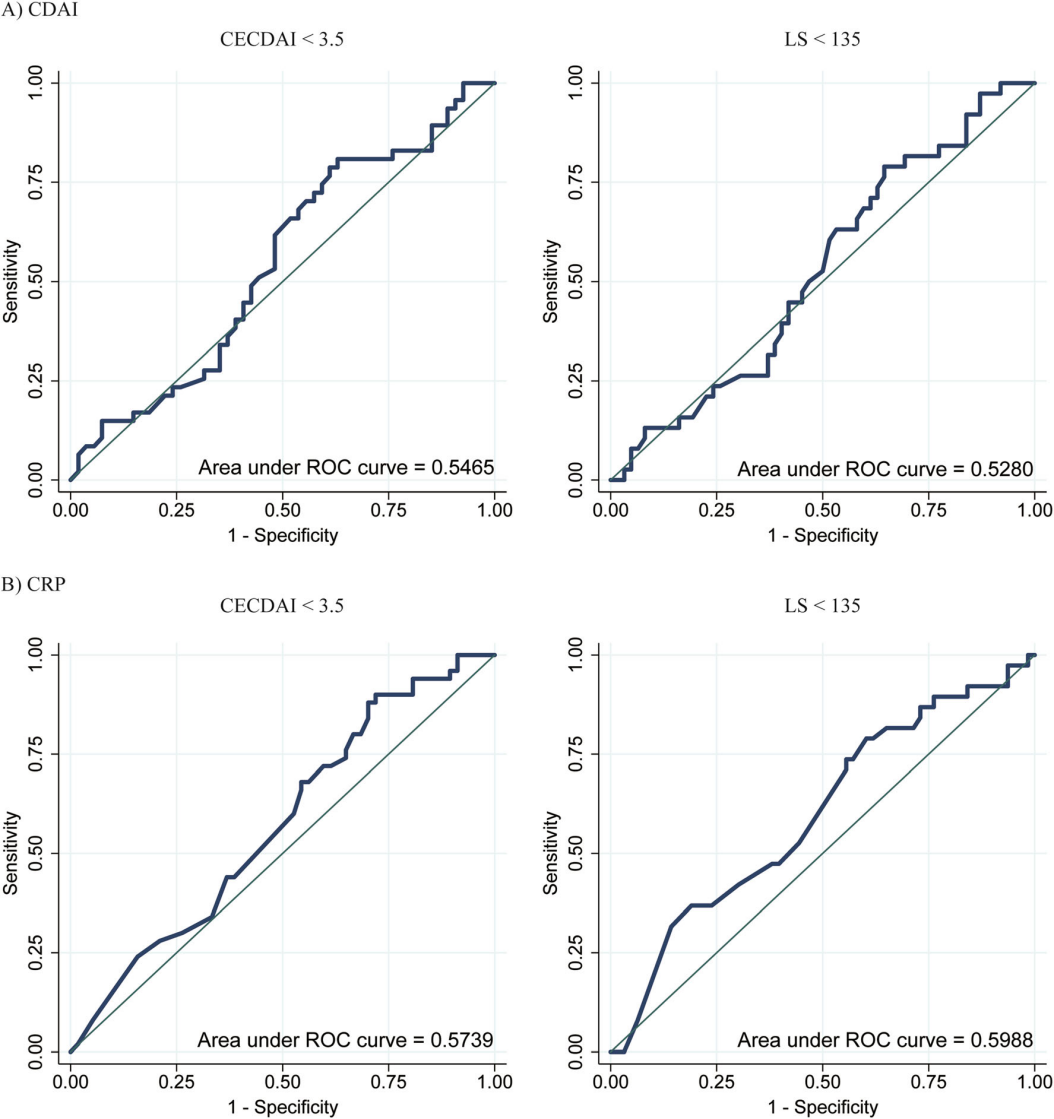
ROC curve of **A** CDAI and **B** CRP for determining CECDAI < 3.5 or LS < 135 in L1 patients with ECD. CDAI and CRP could not demonstrate a high area under the ROC curve. *CDAI* Crohn’s disease activity index, *CECDAI* capsule endoscopy Crohn’s disease activity index, *CRP* C-reactive protein, *ROC* receiver operating characteristics

In patients with L1 (Montreal classification), CDAI < 150 or CRP < 0.15 mg/dL was associated with endoscopic remission, but half of the patients with clinical remission were not under endoscopic remission. For the determination of CECDAI < 3.5, the sensitivity, specificity, PPV, NPV, and accuracy were respectively 0.894, 0.111, 0.467, 0.546, and 0.475 for CDAI < 150 and 0.745, 0.418, 0.522, 0.657, and 0.569 for CRP < 0.15 mg/dL (Table 5). For the determination of LS < 135, the sensitivity, specificity, PPV, NPV, and accuracy were respectively 0.921, 0.129, 0.393, 0.727, and 0.430 for CDAI < 150 and 0.714, 0.413, 0.439, 0.743, and 0.545 for CRP < 0.15 mg/dL. ROC curves of CDAI and CRP for the determination of CECDAI < 3.5 or LS < 135 are shown in Fig. 2. Approximately 32.8–62.6% of patients with CRP < 0.15 mg/dL or CDAI < 150 had CD lesions in the small intestine (jejunum or ileum) (Supplementary Table 3).

**Table 5:** Association of endoscopic and clinical remission in L1 patients; (A) CECDAI and (B) LS

(A) CECDAI								
	CECDAI < 3.5 (*N* = 47)	CECDAI ≥ 3.5 (*N* = 55)	*P* value	Sensitivity	Specificity	PPV	NPV	Accuracy
CDAI	*n* = 47	*n* = 54						
< 150	42 (89.4)	48 (88.9)	< 0.001^†^	0.894	0.111	0.467	0.546	0.475
≥ 150	5 (10.6)	6 (11.1)						
CRP								
< 0.15 mg/dL	35 (74.5)	32 (58.2)	0.003^†^	0.745	0.418	0.522	0.657	0.569
≥ 0.15 mg/dL	12 (25.5)	23 (41.8)						

(B) LS								
	LS < 135 (*N* = 38)	LS ≥ 135 (*N* = 63)	*P* value	Sensitivity	Specificity	PPV	NPV	Accuracy
CDAI	*n* = 38	*n* = 62						
< 150	35 (92.1)	54 (87.1)	< 0.001^†^	0.921	0.129	0.393	0.727	0.430
≥ 150	3 (7.9)	8 (12.9)						
CRP								
< 0.15 mg/dL	29 (76.3)	37 (58.7)	< 0.001^†^	0.714	0.413	0.439	0.743	0.545
≥ 0.15 mg/dL	9 (23.7)	26 (41.3)						

*CECDAI* capsule endoscopy Crohn’s disease activity index, *LS* Lewis score, *CDAI* Crohn’s Disease Activity Index, *CRP* C-reactive protein, *WBC* white blood cell, *SD* standard deviation, *Hb* hemoglobin, *PLT* platelet, *Alb* albumin, *TC* total cholesterol, *IQR* interquartile range, *ESR* erythrocyte sedimentation rate

^†^McNemar test

The rate of a successful evaluation did not differ among the three grades of CD activity, though the rate was relatively high in patients with clinical remission (Supplementary Table 4).

In the patient questionnaire on SBCE experience, 97.1% of patients answered “none” or “almost none” regarding pain, 90.0% answered “none” or “almost none” regarding embarrassment, 14.4% answered “somewhat fearful” or “quite fearful” regarding fear, and 7.0% answered “difficult to swallow” or “very difficult to swallow” regarding swallowing the SBCE (Supplementary Table 5).

## Discussion

In this multicenter study, SBCE was extremely useful for assessing the endoscopic activity of patients with d-CD. In comparison with BAE and MRE, SBCE and BAE have equivalent ability to monitor mucosal activity. MRE was inferior due to a lower detection rate of aphtha or erosion, whereas SBCE was inferior to detect longitudinal ulcers than MRE. The rate of successful evaluation was the highest in patients with clinical remission. Thus, according to the invasiveness of BAE, SBCE is more useful to monitor endoscopic activity in patients with CD, especially with a lower activity or clinical remission, which is supposed to have only small lesions in the small intestine. In addition, SBCE contributed to confirming or excluding CD in patients with s-CD in about 57% of cases. However, most of the undiagnosed cases corresponded to ‘suspicious CD,’ which has aphtha or ulceration in the small intestine but does not fulfill the Japanese diagnostic criteria for CD. There were only a few adverse events and all were related to retention of the SBCE, showing high safety of SBCE in patients with CD. The SBCE incompletion rate (6.5%) was relatively lower than that previously reported [16].

Our results demonstrated the efficacy and safety of SBCE in an unprecedentedly large sample of patients with CD. Notably, SBCE was effective in almost all cases of d-CD. Previously, SBCE was used to monitor small intestinal disease activity in 72% of 74 patients with d-CD [25]. The feasibility of sequential SBCE in patients with d-CD has also been studied [26]. These reports demonstrated the usefulness of SBCE in patients with d-CD; however, the sample sizes were small. Our study showed favorable efficacy in a large sample for the first time.

A meta-analysis reported that SBCE retention occurred in 8% and 4% of patients with d-CD and s-CD, respectively [27]. However, the current study demonstrated a significantly lower retention rate. We believe that this is due to the high frequency of PC tests, which demonstrates their usefulness. In this study, of seven patients with retention, one did not have PC, one had PC but misjudged patency, and the rest of the 5 cases were confirmed patency using PC test. Thus, we did not completely extinguish the risk of retention, but we can reduce the risk using PC test and an appropriate judgment of patency. It was also assumed that physicians avoided SBCE in patients with apparently active CD before implementing the PC test.

However, the diagnostic value in patients with s-CD was lower than that described in a previous report [28]. Despite the presence of aphthous lesions and ulcers in the small intestine, there are many cases in which ‘confirmed CD’ was not diagnosed and remained ‘suspicious CD’. This was due to a lack of longitudinal ulcers or cobblestone appearance, which are essential in Japanese diagnostic criteria for CD. The prevalence of findings in patients with s-CD was 57%, and the ratio of obtaining a CD diagnosis or excluding CD was also 57%. CD is strongly suspected when patients have more than three erosions in the small intestine without taking non-steroidal anti-inflammatory drugs (NSAIDs) [29]. When applying this criterion, the diagnostic value in our study is approximately 60%, the same as a previous report [30].

In Japan, on the other hand, the prevalence of definitive findings, such as a longitudinal ulcer or cobblestone appearance, in patients with s-CD was reported to be approximately 40% [28], which was higher than the 15.7% in our study. Though the difference in the pre-test probabilities is reflected in this result, a lower ability to detect longitudinal ulcers is one of the weak points of SBCE. Whether ‘suspicious CD’ cases are later diagnosed as ‘confirmed CD’ is unclear and should be confirmed, as this might be a clue for detecting early CD [29]. Furthermore, up-to-date diagnostic criteria for CD, including SBCE findings, should be discussed. At present, because the specificity was low in contrast to the high sensitivity, the combined use of SBCE with other modalities with high specificities, such as ileo-colonoscopy or cross-sectional imaging, is considered to increase the definitive diagnostic ratio for CD. In addition, SBCE is suitable for excluding CD even if the pre-examination probability is not very high.

SBCE incompletion contributed to a failure to assess the endoscopic disease activity of d-CD. The factors related to SBCE incompletion were male sex and the presence of stenosis. Risk factors related to SBCE retention were elevated CRP levels and the presence of stenosis. This confirms the presence of a stenotic lesion that can cause poor passage of SBCE as a guide for determining the appropriateness of the examination. Thus, cross-sectional imaging, such as MRE or CTE, is suitable for patients with suspected stenosis or clinically vigorous activity, especially among male patients. With lesions where cross-sectional imaging indicates the presence of active and scarring stenosis, it is better to prioritize other modalities.

This study showed that lesions identified by SBCE were more common in the ileum than in the jejunum. This result is supported by Esaki et al. on the typical distribution of small intestinal lesions in patients with CD [31]. The study demonstrated that the ileum was the most common site for intestinal lesions in patients with CD. Moreover, our results showed that very few patients had CD lesions only in the jejunum. It cannot be concluded that ileo-colonoscopy is sufficient, but it demonstrates that the ileum is the most important site for evaluating small bowel lesions in CD.

Clinical activity indicators and blood examination results were not correlated with SBCE findings, therefore, we analyzed only L1 patients to eliminate colonic inflammation. As a result, half of the patients with CDAI < 150 and CRP < 0.15 mg/dL showed active lesions in the small intestine. CDAI and CRP are champion indices that are used worldwide and in many clinical studies. However, several recent reports have demonstrated that CDAI and CRP levels do not correlate with intestinal activity in patients with lower CD activity [25, 32, 33]. The current study also showed that it is challenging to determine small intestinal mucosal healing using the CDAI or CRP. This means that, even in clinical remission, it is desirable to assess the small intestinal mucosa through endoscopy, such as BAE or SBCE, or to use more sensitive biomarkers. This study did not include an evaluation of fecal calprotectin or leucine-rich alfa 2 glycoprotein, and evidence for these novel markers is needed.

Patient acceptance of SBCE was sufficient. This study shows that SBCE was regarded as minimally invasive and tolerable in patients with CD. Furthermore, the results from the questionnaire supported that SBCE is the best painless method to assess the small intestine.

The limitations of this study are that each facility selected the indications for SBCE without defining clear criteria. A reader performed the interpretation at each facility and the differences between the contrast doctors cannot be adjusted. In addition, selection bias may have occurred due to physicians enrolling few patients with moderate to severe illness. Patients with signs and symptoms of relatively serious CD activity, such as severe abdominal pain and high CRP, were not enrolled in this study because modalities other than SBCE may be more suitable. These factors should be considered when interpreting the results of this study.

In conclusion, SBCE showed high usefulness, safety, and tolerability for assessing the disease activity of patients with lower disease activity or clinical remission of CD. The most suitable use of SBCE is to monitor small-intestinal lesions, especially in patients who have achieved clinical remission after treatment. For patients with s-CD, it is desirable to use SBCE in combination with colonoscopy or cross-sectional imaging. SBCE is also considered highly useful in excluding CD.

### Supplementary Information

Below is the link to the electronic supplementary material.

Supplementary Fig. 1 Flow diagram of enrolled patients. Of the 544 patients analyzed, 541 underwent SBCE. Of these patients, 93.5% completed SBCE. Abbreviations: PC patency capsule, SBCE small bowel capsule endoscopy. (PDF 432 kb)

Supplementary file2 (DOCX 31 kb)
