# Supplementary figures and images for: Multicenter prospective registration study of efficacy and safety of capsule endoscopy in Crohn’s disease in Japan (SPREAD-J study)
Source: J Gastroenterol. 2023 Jul 21;58(10):1003–14. doi: 10.1007/s00535-023-02017-3 (PMC10522504; doi:10.1007/s00535-023-02017-3)

Supplementary Fig. 1 Flow diagram of enrolled patients

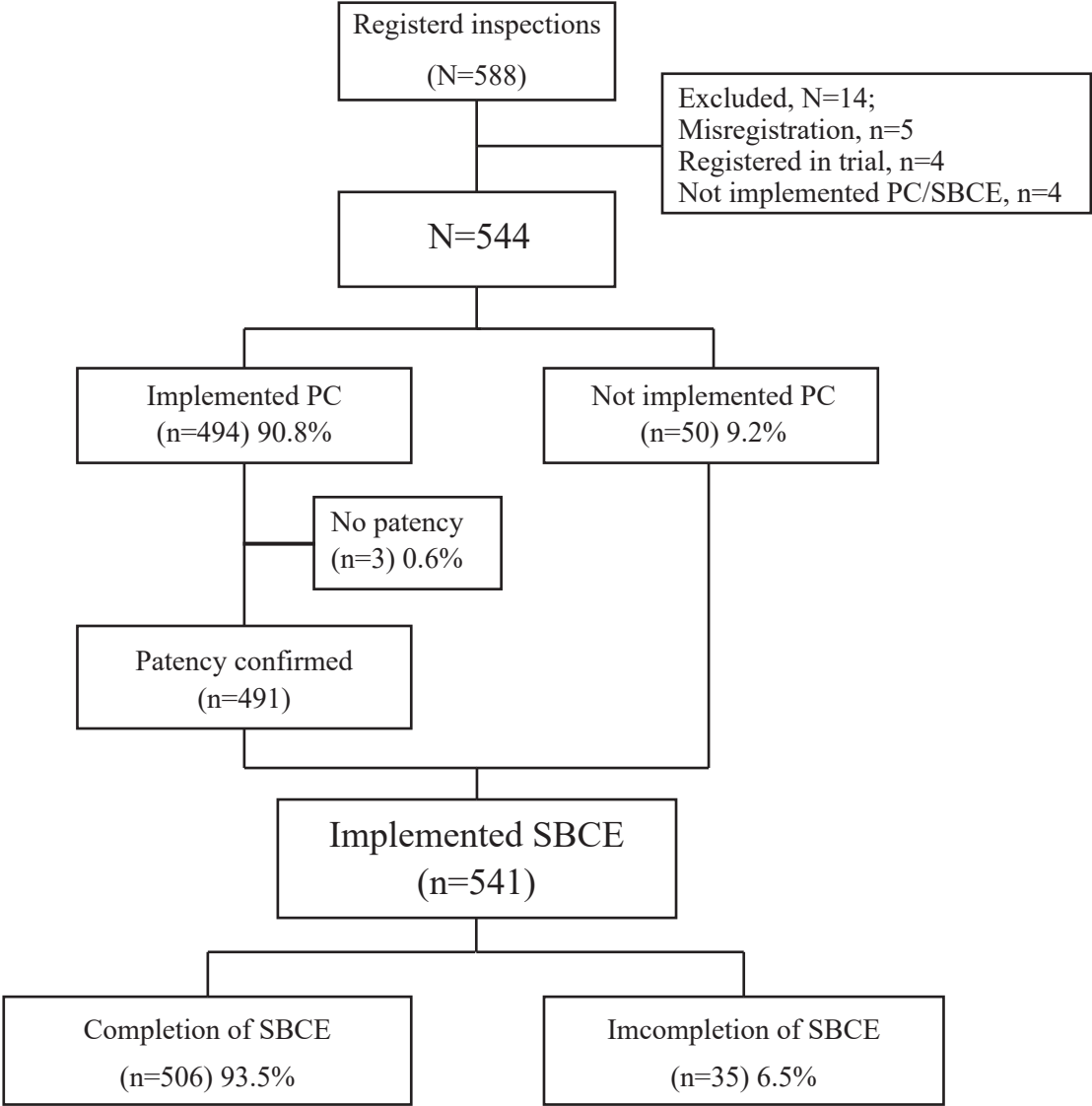

Supplement: Supplementary file 1 — Supplementary Fig. 1 Flow diagram of enrolled patients. Of the 544 patients analyzed, 541 underwent SBCE. Of these patients, 93.5% completed SBCE. Abbreviations: PC patency capsule, SBCE small bowel capsule endoscopy. (PDF 432 kb) [file 535_2023_2017_MOESM1_ESM.pdf]
