# Supplementary material for: Multicenter prospective registration study of efficacy and safety of capsule endoscopy in Crohn’s disease in Japan (SPREAD-J study)
Source: J Gastroenterol. 2023 Jul 21;58(10):1003–14. doi: 10.1007/s00535-023-02017-3 (PMC10522504; doi:10.1007/s00535-023-02017-3)
Supplement: Supplementary file 2 — Supplementary file2 (DOCX 31 kb) [file 535_2023_2017_MOESM2_ESM.docx]

**Supplementary materials**

**Supplementary Table 1.** Diagnostic criteria for Crohn’s disease in Japan^17,18^

| **Main findings** |
| --- |
| A. Longitudinal ulcer (in the case of the small intestine, preferably on the mesenteric attachment) |
| B. Cobble stone appearance |
| C. Noncavitary epithelioid cell granuloma: serial sectioning of histology samples improves the diagnostic yield. The diagnosis should be made by a pathologist familiar with the gastrointestinal tract |
| **Secondary findings** |
| a. Extensive irregular to round ulcers or aphthae in the gastrointestinal tract: extensive gastrointestinal tract lesions mean that the lesions are anatomically distributed over more than one organ, i.e., the upper gastrointestinal tract (esophagus, stomach, duodenum), small intestine, and large intestine. The lesions are typically longitudinal but may not be longitudinal. The disease should be permanent for at least three months. Multiple rings may be in the Kerckring folds of the duodenum and small intestine on capsule endoscopy. It is necessary to exclude intestinal tuberculosis, intestinal Behçet's disease, simple ulcer, nonsteroidal anti-inflammatory drug ulcers, and infectious enteritis |
| b. Characteristic anorectal lesions: anal fissures, cavitating ulcers, hemorrhoids, perianal abscesses, edematous cortices, etc. We recommend that physicians ask an anorectologist familiar with Crohn’s disease and use the Crohn’s Disease Atlas of Anorectal Lesions to confirm the diagnosis |
| c. Characteristic gastric and duodenal lesions: bamboo joint-like appearance, notch-like depressions. A specialist in Crohn’s disease should make the diagnosis |
| **Confirmed diagnosis of Crohn’s disease** |
| 1. Patients with main findings A or B. In the case of only a longitudinal ulcer, ischemic bowel disease or ulcerative colitis should be excluded. In the case with only a cobblestone appearance is present, ischemic bowel lesions and type 4 colorectal cancer should be excluded |
| 1. Main finding of C with the secondary finding of a or b |
| 1. Patients with all the secondary findings (a, b, and c) |
| Note 1: Inflammatory bowel disease unclassified may develop more characteristic features of one of these diseases with follow-up |
| **Suspicious diagnosis of Crohn’s disease** |
| 1. Main finding of C, with the secondary finding of c |
| 1. Main finding A or B, but cannot be differentiated from ischemic colitis or ulcerative colitis |
| 1. Main finding of C only |
| 1. One or two secondary findings |

**Supplementary Table 2.** Characteristics of eligible patients

|  | Total | d-CD | s-CD | p-value |
| --- | --- | --- | --- | --- |
| Patients, n (%) | 544 | 468 (86.0) | 76 (14.0) |  |
| Sex, male, n (%) | 379 (69.7) | 338 (72.2) | 41 (53.9) | 0.001 |
| Age, years, mean ± SD | 34.8 ± 13.5 | 34.7 ± 13.4 | 35.3 ± 14.3 | 0.838 |
| Montreal Classification |  |  |  |  |
| L1, n (%) |  | 102 (21.8) |  |  |
| L2, n (%) |  | 29 (6.2) |  |  |
| L3, n (%) |  | 334 (71.4) |  |  |
| L4, n (%) |  | 8 (1.7) |  |  |
| Disease period, months, median (IQR) | 77 (28-150) | 83.5 (35-163) | 10 (2-65) | <0.001 |
| History of intestinal resection, positive, n (%) | 391 (71.9) | 148 (31.6) | 4 (5.3) | <0.001 |
| Ileostomy, positive, n (%) | 34 (6.3) | 34 (7.2) |  |  |
| Concomitant drug |  |  |  |  |
| 5-Aminosalicylic acid, n (%) | 365 (67.1) | 345 (73.7) | 20 (26.3) | <0.001 |
| Prednisolone, n (%) | 14 (2.6) | 11 (2.4) | 3 (3.9) | 0.415 |
| Immunomodulators, n (%) | 140 (25.7) | 136 (29.1) | 4 (5.3) | <0.001 |
| Anti-TNF-α antibody, n (%) | 248 (45.6) | 247 (52.8) | 1 (1.3) | <0.001 |
| Infliximab, n (%) | 131 (24.1) | 130 (27.8) | 1 (1.3) | <0.001 |
| Adalimumab, n (%) | 114 (21.0) | 114 (24.4) | 0 (0.0) | <0.001 |
| Ustekinumab, n (%) | 50 (9.2) | 50 810.7) | 0 (0.0) | 0.003 |
| Vedolizumab, n (%) | 4 (0.7) | 4 (0.9) | 0 (0.0) | 0.419 |
| Elementary diet, n (%) | 64 (11.8) | 63 (13.5) | 1 (1.3) | 0.002 |
| None, n (%) | 59 (10.9) | 7 (1.5) | 52 (68.4) | <0.001 |
| Blood sampling data |  |  |  |  |
| WBC, /μL, mean ± SD | 6,138 ± 2,003 | 6,126 ±1,943 | 6,536 ±2,319 | <0.001 |
| Hb, g/dL, mean ± SD | 13.7 ± 1.9 | 13.7 ±1.8 | 13.4 ±2.0 | 0.415 |
| PLT, 104/μL, mean ± SD | 27.5 ± 8.0 | 27.4 ± 7.7 | 28.2 ±9.5 | 0.048 |
| Albumin, g/dL, mean ± SD | 4.2 ± 0.5 | 4.2 ± 0.5 | 4.2 ±0.5 | 0.567 |
| TC, mg/dL, mean ± SD | 165 ± 37 | 164 ± 36 | 168 ± 37 | <0.001 |
| CRP, mg/dL, median (IQR) | 0.1 (0.03-0.32) | 0.1 (0.03-0.31) | 0.1 (0.03-0.42) | <0.001 |
| ESR, mm, median (IQR) | 11 (5-24) | 11.5 (5-24) | 9 (3-29) | <0.001 |
| CDAI, mean ± SD |  | 83.4 ± 66.7 |  |  |

*d-CD* definitive Crohn’s disease, *s-CD* suspected Crohn’s disease, *IQR* interquartile range, *WBC* white blood cell, *Hb* hemoglobin, *PLT* platelet, *TC* total cholesterol, *CRP* C-reactive protein, *ESR* erythrocyte sedimentation rate, *CDAI* Crohn’s disease activity index, *SD* standard deviation.

**Supplementary Table 3.** SBCE findings in L1 patients with d-CD divided by A) CRP 0.15 mg/dL and B) CDAI 150.

1. CRP

|  | CRP <0.15 mg/dL (N=67) | CRP ≥0.15 mg/dL (N=35) | p-value |
| --- | --- | --- | --- |
| Jejunum |  |  |  |
| Edema | 10 (14.9) | 9 (25.7) | 0.184 |
| Aphthae | 9 (13.4) | 9 (25.7) | 0.122 |
| Erosion | 7 (10.5) | 9 (25.7) | 0.044 |
| Ulcer | 7 (10.5) | 5 (14.3) | 0.568 |
| Longitudinal ulcer | 1 (1.5) | 1 (2.9) | 0.637 |
| Cobblestone appearance | 0 (0.0) | 0 (0.0) | NA |
| Stenosis | 3 (4.5) | 2 (5.7) | 0.866 |
| No findings of CD | 45 (67.2) | 18 (51.4) | 0.121 |
| Ileum |  |  |  |
| Edema | 14 (20.9) | 9 (25.7) | 0.580 |
| Aphthae | 16 (23.9) | 8 (22.9) | 0.908 |
| Erosion | 19 (28.4) | 15 (42.9) | 0.140 |
| Ulcer | 22 (32.8) | 15 (42.9) | 0.523 |
| Longitudinal ulcer | 1 (1.5) | 6 (17.1) | 0.003 |
| Cobblestone appearance | 0 (0.0) | 2 (5.7) | 0.048 |
| Stenosis | 14 (20.9) | 7 (20.0) | 0.881 |
| No findings of CD | 25 (37.3) | 10 (28.6) | 0.377 |

1. CDAI

|  | CDAI <150 (N=90) | CDAI ≥150 (N=11) | p-value |
| --- | --- | --- | --- |
| Jejunum |  |  |  |
| Edema | 17 (18.9) | 2 (18.2) | 0.955 |
| Aphthae | 16 (17.8) | 2 (18.2) | 0.974 |
| Erosion | 13 (14.4) | 3 (27.3) | 0.271 |
| Ulcer | 11 (12.2) | 1 (9.1) | 0.762 |
| Longitudinal ulcer | 1 (1.1) | 1 (9.1) | 0.073 |
| Cobblestone appearance | 0 (0.0) | 0 (0.0) | NA |
| Stenosis | 5 (5.6) | 0 (0.0) | 0.431 |
| No findings of CD | 54 (60.0) | 8 (72.7) | 0.413 |
| Ileum |  |  |  |
| Edema | 20 (22.2) | 3 (27.3) | 0.706 |
| Aphthae | 20 (22.2) | 3 (27.3) | 0.706 |
| Erosion | 28 (31.1) | 5 (45.5) | 0.338 |
| Ulcer | 29 (32.2) | 6 (54.6) | 0.142 |
| Longitudinal ulcer | 4 (4.4) | 3 (27.3) | 0.005 |
| Cobblestone appearance | 1 (1.1) | 1 (9.1) | 0.073 |
| Stenosis | 18 (20.0) | 2 (18.2) | 0.926 |
| No findings of CD | 32 (35.6) | 3 (27.3) | 0.586 |

Data are presented as n (%).

*CD* Crohn’s disease, *d-CD* definitive Crohn’s disease, *CRP* C-reactive protein, *CDAI* Crohn’s disease activity index, *SBCE* small-bowel capsule endoscopy.

|  | clinical activity (n=424) | | |
| --- | --- | --- | --- |
|  | Remission (n=363) | Mild  (n=42) | Moderate  (n=19) |
| Successful evaluation | 359 (98.9) | 41 (97.6) | 18 (90.0) |

**Supplementary Table 4.** A comparison of the rate of successful evaluation among three classifications of clinical activity of CD.

**Supplementary Table 5.** Results of Patient Acceptance of SBCE Questionnaire

| N=518 | None | A little | Some | A lot | No idea | No answer |
| --- | --- | --- | --- | --- | --- | --- |
| Embarrassment | 329 (63.5) | 137 (26.5) | 44 (8.5) | 5 (1.0) | 2 (0.4) | 1 (0.2) |
| Fear | 282 (54.4) | 159 (30.7) | 67 (12.9) | 8 (1.5) | 1 (0.2) | 1 (0.2) |
| Pain | 433 (83.6) | 70 (13.5) | 9 (1.7) | 1 (0.2) | 3 (0.6) | 2 (0.4) |
|  |  |  |  |  |  |  |
|  | Very easy | Easy | Neither | Difficult | Very difficult | No answer |
| Swallowing capsule | 112 (21.6) | 278 (53.7) | 91 (17.6) | 33 (6.4) | 3 (0.6) | 1 (0.2) |
|  |  |  |  |  |  |  |
| Which do you think is more accessible than SBCE? | SBCE is easier | SBCE is a little easier | Neither | SBCE is a little harder | SBCE is harder | No answer |
| Small intestinal BE | 326 (62.9) | 47 (9.1) | 32 (6.2) | 6 (1.2) | 3 (0.6) | 104 (20.1) |
| BAE | 294 (56.8) | 39 (7.5) | 23 (4.4) | 2 (0.4) | 2 (0.4) | 158 (30.5) |
|  |  |  |  |  |  |  |
|  | I prefer | I do not prefer | No idea | No answer |  |  |
| Are you willing to proceed with SBCE next time? | 381 (73.6) | 118 (22.8) | 16 (3.1) | 3 (0.6) |  |  |

*SBCE* small-bowel capsule endoscopy, *BE* barium enema, *BAE* balloon-assisted endoscopy.
